# The Effects of Mesenchymal Stem Cells on Antimelanoma Immunity Depend on the Timing of Their Administration

**DOI:** 10.1155/2020/8842659

**Published:** 2020-07-10

**Authors:** Dragana Miloradovic, Dragica Miloradovic, Bojana Simovic Markovic, Aleksandar Acovic, Carl Randall Harrell, Valentin Djonov, Nebojsa Arsenijevic, Vladislav Volarevic

**Affiliations:** ^1^Center for Molecular Medicine and Stem Cell Research, Department of Microbiology and Immunology, Faculty of Medical Sciences, University of Kragujevac, 69 Svetozar Markovic Street, Kragujevac, Serbia; ^2^Regenerative Processing Plant, LLC, 34176 US Highway 19 N Palm Harbor, Palm Harbor FL, USA; ^3^Institute of Anatomy, University of Bern, 2 Baltzerstrasse, Switzerland; ^4^Center of Excellence for the Acceleration of Harm Reduction (CoEHAR), Università di Catania, Via Santa Sofia 78, Catania 95123, Italy

## Abstract

There is still a lively debate about whether mesenchymal stem cells (MSCs) promote or suppress antitumor immune response. Although several possible explanations have been proposed, including different numbers of injected and engrafted MSCs, heterogeneity in phenotype, and function of tumor cells, the exact molecular mechanisms responsible for opposite effects of MSCs in modulation of antitumor immunity are still unknown. Herewith, we used a B16F10 murine melanoma model to investigate whether timing of MSC administration in tumor-bearing mice was crucially important for their effects on antitumor immunity. MSCs, intravenously injected 24 h after melanoma induction (B16F10+MSC^1d^-treated mice), significantly enhanced natural killer (NK) and T cell-driven antitumor immunity, suppressed tumor growth, and improved survival of melanoma-bearing animals. Significantly higher plasma levels of antitumorigenic cytokines (TNF-*α* and IFN-*γ*), remarkably lower plasma levels of immunosuppressive cytokines (TGF-*β* and IL-10), and a significantly higher number of tumor-infiltrating, IFN-*γ*-producing, FasL- and granzyme B-expressing NK cells, IL-17-producing CD4+Th17 cells, IFN-*γ*- and TNF-*α*-producing CD4+Th1 cells, and CD8+cytotoxic T lymphocytes (CTLs) were observed in B16F10+MSC^1d^-treated mice. On the contrary, MSCs, injected 14 days after melanoma induction (B16F10+MSC^14d^-treated mice), promoted tumor growth by suppressing antigen-presenting properties of tumor-infiltrating dendritic cells (DCs) and macrophages and by reducing tumoricidal capacity of NK cells and T lymphocytes. Significantly higher plasma levels of TGF-*β* and IL-10, remarkably lower plasma levels of TNF-*α* and IFN-*γ*, and significantly reduced number of tumor-infiltrating, I-A-expressing, and IL-12-producing macrophages, CD80- and I-A-expressing DCs, granzyme B-expressing CTLs and NK cells, IFN-*γ*- and IL-17-producing CTLs, CD4+Th1, and Th17 cells were observed in B16F10+MSC^14d^-treated animals. In summing up, the timing of MSC administration into the tumor microenvironment was crucially important for MSC-dependent modulation of antimelanoma immunity. MSCs transplanted during the initial phase of melanoma growth exerted tumor-suppressive effect, while MSCs injected during the progressive stage of melanoma development suppressed antitumor immunity and enhanced tumor expansion.

## 1. Introduction

Melanoma is nowadays considered as one of the most aggressive and the fastest growing malignant tumors worldwide [1]. Although a primary cutaneous melanoma can be managed by surgery, the advanced metastatic melanoma requires use of modern molecular mechanism-based therapeutic approaches [1]. The immuno- and targeted drug therapies, which interfere with oncoprotein and immune checkpoint pathways, were able to positively impact survival of patients with advanced melanoma [2]. Unfortunately, the success rate is being hampered by a number of factors including drug resistance, heterogeneous phenotype of melanoma cells, and impaired activation of antitumor immune response [2]. Therefore, new and more effective strategies are needed for patients who did not receive optimal benefit from currently used therapeutic approaches.

Mesenchymal stem cells (MSCs) are nonhematopoietic, multipotent stem cells that reside in almost all postnatal tissues [3]. As cells of mesodermal origin, MSCs are considered as an integral part of the tumor stromal microenvironment, where, together with malignant cells, fibroblasts, pericytes, and endothelial cells, it produces trophic and growth factors and immuno- and angiomodulatory molecules and regulates tumor development [4]. Additionally, MSCs express a large number of chemokine receptors and exhibit strong tropism towards cancer cells [5]. After systemic administration, MSCs engraft in the tumor microenvironment where, in a juxtacrine and paracrine manner, it regulates expansion of malignant cells and modulates antitumor immunity [6]. Due to their tumor-homing capacity, MSCs were used as a vehicle to deliver cytotoxic drugs, proinflammatory cytokines, and cell cycle-interfering microRNAs in the tumors, attenuating their growth and progression [7]. MSCs modulate phenotype and function of all immune cells that play an important role in antitumor immune response [8]. MSCs regulate antigen-presenting properties of macrophages and dendritic cells (DCs), cytotoxicity of natural killer (NK) and CD8+T cells (CTLs), and cytokine production in CD4+T helper cells [8]. Accordingly, effects of MSC-dependent modulation of antitumor immunity have been explored in a large number of experimental studies, but surprisingly, opposite results were reported. While several research groups demonstrated that MSCs suppressed antitumor immune response and enhanced tumor progression [9–11], experimental findings presented by other researchers indicated that MSC-based therapy favored development of strong antitumor immunity that inhibited expansion of malignant cells [9–13]. Although several possible explanations for these contradictory findings have been proposed, including different numbers of injected and engrafted MSCs, diverse route of their administration, heterogeneity in phenotype, and function of tumor cells [12, 13], the exact molecular mechanisms responsible for opposite effects of MSCs in modulation of tumor growth are still unknown. Recently, Zong and colleagues indicated that MSC-based effects on progression and metastasis of hepatocellular carcinoma (HCC) depended on the time of MSC administration in the tumor-bearing animals [14]. Injection of MSCs in the initial phase of HCC development resulted in tumor suppression, while MSCs administered in the progressive stage of tumor growth promoted progression and metastasis of HCC [14]. In line with these findings, herewith, we used a murine model of melanoma to investigate whether the timing of MSC administration in melanoma-bearing mice was crucially important for MSC-dependent modulation of antitumor immunity and melanoma progression.

## 2. Material and Methods

### 2.1. Cells

MSCs isolated from bone marrow of C57BL/6 mice were purchased from Gibco (Catalog number S1502-100). The murine melanoma cell line B16F10, which is syngeneic to the C57BL/6 background, was purchased from the American Type Culture Collection (CRL-6475; ATCC, USA). Both types of cells were cultured in complete Dulbecco's modified Eagle medium (DMEM) containing 10% heat-inactivated fetal bovine serum (FBS), 100 IU/mL penicillin G, and 100 *μ*g/mL streptomycin (Sigma-Aldrich, Munich, Germany), at 37°C in a 5% CO_2_ incubator. MSCs in passage 4 and B16F10 cells in passage 4 were used throughout the experiments.

### 2.2. Animals

Eight- to ten-week-old C57BL/6 mice were used. Mice were maintained in animal breeding facilities of the Faculty of Medical Sciences, University of Kragujevac, Serbia. All procedures were performed in accordance with the guidelines for the Principles of Laboratory Animal Care and the *Guide for the Care and Use of Laboratory Animals*, and all animals received humane care according to the criteria outlined in the *Guide for the Care and Use of Laboratory Animals* (National Institutes of Health publication 86-23, 1985 revision). All experiments were approved by the Animal Ethical Review Board of the Faculty of Medical Sciences, University of Kragujevac, Serbia. Mice were housed in a temperature-controlled environment with a 12-hour light-dark cycle and were administered with standard laboratory chow and water *ad libitum*. At least eight mice per group were used in each experiment.

### 2.3. Melanoma Induction and Injection of MSCs

B16F10 cells (5 × 10^5^ cells, suspended in 200 *μ*L of phosphate-buffered saline (PBS)) were subcutaneously injected in the left flank of C57BL/6 mice. Immediately after, mice were divided into four experimental groups. The first experimental group of mice, 1 day after injection of B16F10 cells, intravenously received MSCs (5 × 10^5^ cells, suspended in 200 *μ*L of PBS; B16F10+MSC^1d^-treated mice). The second experimental group of B16F10-treated animals, 14 days after administration of B16F10 cells, intravenously received MSCs (5 × 10^5^ cells, suspended in 200 *μ*L of PBS; B16F10+MSC^14d^-treated mice). Mice from the third and fourth experimental groups intravenously received 200 *μ*L of PBS at appropriate time points (1 day (B16F10+PBS^1d^-treated mice) or 14 days after B16F10 administration (B16F10+PBS^14d^-treated animals)). All animals were sacrificed 28 days after the injection of B16F10 cells.

### 2.4. Measurement of Tumor Growth and Progression

Once the tumors were palpable, they were measured daily and tumor volume was calculated with the following formula: *V* = 4/3*π*∗*a*/2∗*b*/2∗*c*/2 (*a* = length, *b* = width, and *c* = thickness) [15].

### 2.5. Measurement of Cytokines in Plasma Samples of Tumor-Bearing Mice

Blood samples were collected from the facial vein at days 1, 14, and 28 after the injection of B16F10 cells. Mouse blood was kept in anticoagulant-containing tubes and centrifuged for 10 minutes at 2000 g at 4°C. Supernatants were stored at -20°C until needed. Concentration of tumor necrosis factor alpha (TNF-*α*), interferon gamma (IFN-*γ*), transforming growth factor beta (TGF-*β*), and interleukin- (IL-) 10 in mouse plasma samples were measured by using enzyme-linked immunosorbent assay (ELISA) sets (R&D Systems, Minneapolis, MN, USA), according to the manufacturer's instructions [16].

### 2.6. Isolation of Tumor-Infiltrating Leucocytes

By using forceps and scissors, subcutaneous tumors were resected *en bloc*, including overlying and surrounding skin. After the removal of surrounding skin, tumors were measured and weighed. By using scissors, the tumors were minced, until all large sections were processed into 1-2 mm pieces which are digested in 5 mL of DMEM containing 1 mg/mL collagenase I, 1 mM EDTA, and 2% FBS (all from Sigma-Aldrich, Munich, Germany). After incubation of 2 hr at 37°C, the digested tumor tissue was incubated with 4 mL of trypsin and DNase I (Roche Diagnostics), followed by passing through a 40 *μ*m nylon filter. Single-cell suspensions were then processed for flow cytometry analysis [17].

### 2.7. Flow Cytometry Analysis and Intracellular Staining of Tumor-Infiltrating Leucocytes

Tumor-infiltrating leucocytes were investigated for different cell surface and intracellular markers with flow cytometry. Briefly, 1 × 10^6^ cells were incubated with anti-mouse F4/80, CD4, CD8, CD11c, NK1.1, CD80, I-A, granzyme B, and Fas ligand (FasL) monoclonal antibodies conjugated with fluorescein isothiocyanate (FITC), phycoerythrin (PE), peridinin chlorophyll protein (PerCP), or allophycocyanin (APC) (all from BD Biosciences, San Jose, CA, USA) following the manufacturer's instructions. Immune cells derived from the tumors were concomitantly stained for the intracellular content of TNF-*α*, IFN-*γ*, IL-12, IL-4, and IL-17 by using the fixation/permeabilization kit and anti-mouse monoclonal antibodies conjugated with FITC, PE, PerCP, and APC (BD Biosciences). For intracellular cytokine staining, cells were stimulated with 50 ng/mL PMA and 500 ng/mL ionomycin for 5 h, and GolgiStop (BD Biosciences) was added. Cells were fixed in Cytofix/Cytoperm, permeated with 0.1% saponin, and stained with fluorescent Abs. Flow cytometric analysis was conducted on a BD Biosciences' FACSCalibur and analyzed by using the Flowing Software analysis program [17].

### 2.8. Statistical Analyses

The data were analyzed using statistical package SPSS, version 21. The normality of distribution was tested by the Kolmogorov-Smirnov test. The results were analyzed using the Student t-test. All data in this study were expressed as the mean ± standard error of the mean (SEM). Values of p < 0.05 were considered as statistically significant.

## 3. Results

### 3.1. MSC-Based Modulation of Melanoma Growth Depends on the Time of MSC Administration

**Figure 1:**
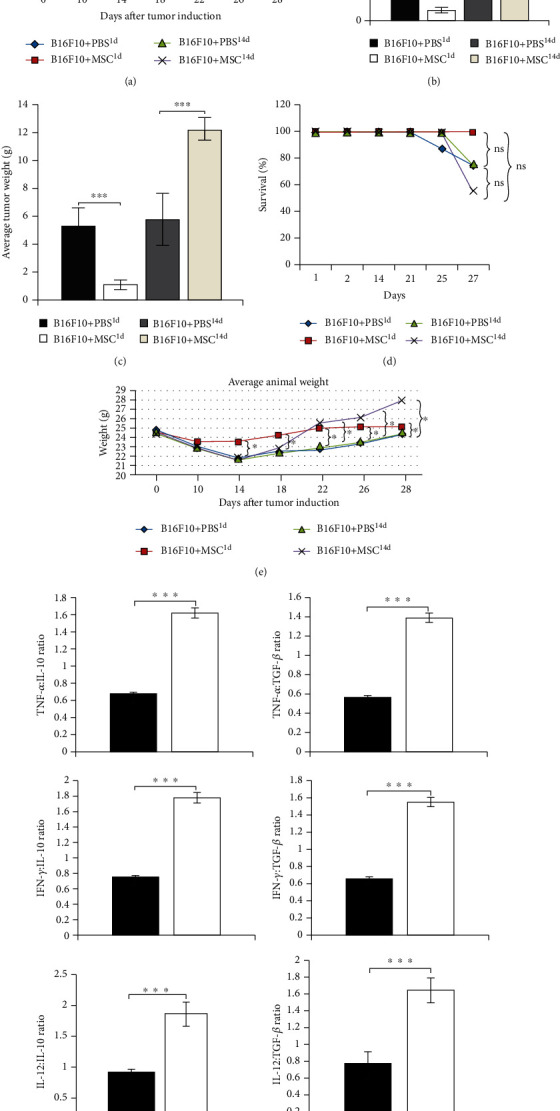
MSC-based modulation of melanoma growth depends on the time of MSC administration. Delayed tumor growth, observed in B16F10+MSC^1d^-treated mice, and rapid melanoma growth, noticed in B16F10+MSC^14d^-treated animals from day 18, were evidenced by the measurement of tumor volumes at different days after tumor induction (a). Significantly lower average tumor volume (b) and tumor weight (c) were observed in B16F10+MSC^1d^-treated mice than in B16F10+PBS^1d^-treated animals at day 28. Oppositely, average tumor volume (b) and tumor weight (c) were significantly greater in B16F10+MSC^14d^-treated mice than in B16F10+PBS^14d^-treated animals at day 28. The lowest survival rate was noticed in B16F10+MSC^14d^-treated animals, while all of B16F10+MSC^1d^-treated mice survived to the last, 28^th^ day of experiment (d). The difference in the survival between experimental groups was statistically nonsignificant (“ns”). Average animal weight at different days after tumor induction demonstrates reduced weight loss in MSC-treated, melanoma-bearing mice (e). The ratios of proinflammatory to anti-inflammatory cytokines (TNF-*α* : IL-10, TNF-*α* : TGF-*β*, IFN-*γ* : IL-10, IFN-*γ* : TGF-*β*, IL-12 : IL-10, and IL-12 : TGF-*β*) were significantly lower in plasma samples of B16F10+PBS^1d^-treated mice than in plasma samples of B16F10+PBS^14d^-treated animals (f). Plasma samples were collected 24 h and 14 days after tumor induction. Values are presented as the mean ± SEM; *n* = 8 mice/group. ^∗^*p* < 0.05, ^∗∗∗^*p* < 0.001.

First, we examined whether systemic application of MSCs affected melanoma growth. As it is shown in Figure 1(a), tumors become palpable in B16F10+MSC^1d^-treated mice 8 days later compared with other experimental groups, suggesting that MSCs, intravenously injected 24 h after melanoma induction, prevented rapid tumor growth. Starting from day 18, average tumor volumes were significantly lower in B16F10+MSC^1d^-treated mice than in B16F10+PBS^1d^-treated animals (*p* < 0.05; Figure 1(a)). Additionally, the average volume and weight of tumors removed from B16F10+MSC^1d^-treated mice at day 28 were significantly lower than melanomas taken from B16F10+PBS^1d^-treated animals (Figures 1(b) and 1(c)), confirming that MSCs, intravenously injected 24 h after melanoma induction, efficiently suppressed tumor growth and progression.

Opposite to these data were results observed in melanoma-bearing animals that intravenously received MSCs 14 days after tumor induction (B16F10+MSC^14d^-treated mice). Starting from day 18 (4 days after MSC injection), average tumor volumes were significantly greater in B16F10+MSC^14d^-treated animals than in B16F10+PBS^14d^-treated mice (*p* < 0.05; Figure 1(a)). Accordingly, at day 28, average volume and weight of tumor removed from B16F10+PBS^14d^-treated mice were significantly lower than those of melanomas of B16F10+MSC^14d^-treated animals (Figures 1(b) and 1(c)), confirming that MSCs administered 14 days after tumor induction remarkably enhanced melanoma growth and progression. In line with these findings, the time of MSC injection was crucially important for their effects on survival of melanoma-bearing mice. While the lowest survival rate was observed in B16F10+MSC^14d^-treated mice, all of the melanoma-bearing animals that received MSCs 24 h after tumor induction survived till the end of the experiment (Figure 1(d)).

Starting from day 14, MSCs transplanted 24 h after tumor induction significantly reduced weight loss of melanoma-bearing mice (*p* < 0.05; Figure 1(e)). Interestingly, weight gain was also noticed in B16F10+MSC^14d^-treated animals (*p* < 0.05; Figure 1(e)). While reduced weight of B16F10+MSC^1d^-treated mice could be contributed to the MSC-dependent suppression of tumor progression, weight gain, noticed in B16F10+MSC^14d^-treated animals, may be a consequence of significantly increased tumor weight which was observed in these mice.

Since MSCs adopt proinflammatory (MSC1) or immunosuppressive (MSC2) phenotype in response to the inflammatory and immunosuppressive cytokines to which they are exposed [18], we analyzed and compared the concentration of inflammatory (TNF-*α*, IFN-*γ*) and immunosuppressive cytokines (IL-10, TGF-*β*) in plasma samples of melanoma-bearing mice at the time of MSC administration. The ratios of proinflammatory to anti-inflammatory cytokines (TNF-*α* : IL-10, TNF-*α* : TGF-*β*, IFN-*γ* : IL-10, IFN-*γ* : TGF-*β*, IL-12 : IL-10, and IL-12 : TGF-*β*) were significantly lower in plasma samples of B16F10+PBS^1d^-treated mice compared to B16F10+PBS^14d^-treated animals (*p* < 0.001; Figure 1(d)), suggesting that MSCs, administered 1 day after the injection of tumor cells, were exposed to the higher concentration of immunosuppressive cytokines, while MSCs transplanted 14 days after tumor induction were exposed to the higher concentration of inflammatory cytokines. Therefore, we assume that, in response to the different concentration of inflammatory and immunosuppressive cytokines to which they were exposed, MSCs injected during the initial phase of melanoma growth adopted proinflammatory (MSC1) phenotype, while MSCs that were transplanted during the progressive stage of melanoma development adopted immunosuppressive (MSC2) phenotype.

### 3.2. MSCs, Injected 24 h after Melanoma Induction, Significantly Enhanced NK and T Cell-Driven Antitumor Immunity and Suppressed Tumor Growth and Progression

**Figure 2:**
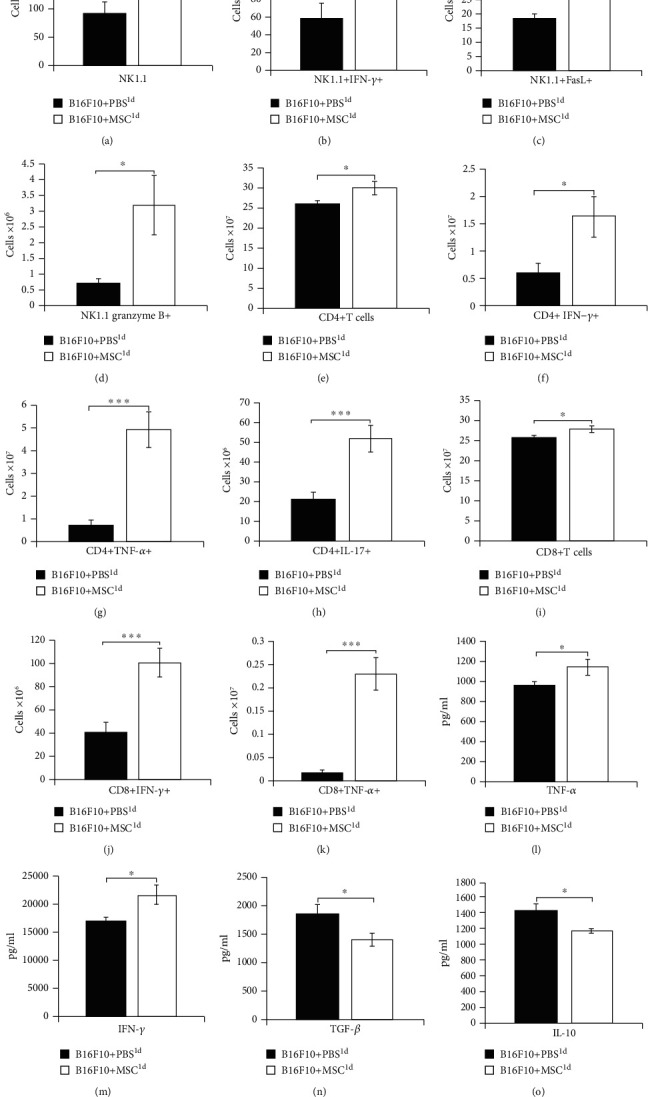
MSCs, injected 24 h after melanoma induction, significantly enhanced NK and T cell-driven antitumor immunity and suppressed tumor growth and progression. Significantly higher number of NK1.1+NK cells (a), IFN-*γ*-producing NK cells (b), FasL- and granzyme B-expressing NK cells (c, d), CD4+T cells (e), IFN-*γ*- and TNF-*α*-producing CD4+Th1 cells (f, g), IL-17-producing CD4+Th17 cells (h), CD8+CTLs (i), and IFN-*γ*- and TNF-*α*-producing CD8+CTLs (j, k) were noticed in the tumors of B16F10+MSC^1d^-treated mice compared to the B16F10+PBS^1d^-treated animals. Significantly higher concentration of inflammatory and antitumorigenic cytokines TNF-*α* and IFN-*γ* (l, m) and significantly lower concentration of immunosuppressive cytokines TGF-*β* and IL-10 (n, o) were noticed in plasma samples of B16F10+MSC^1d^-treated mice compared to B16F10+PBS^1d^-treated animals. Values are presented as the mean ± SEM; *n* = 8 mice/group. ^∗^*p* < 0.05, ^∗∗∗^*p* < 0.001.

Cellular makeup of tumors obtained from B16F10+PBS^1d^- and B16F10+MSC^1d^-treated mice revealed that MSCs, injected 24 h after melanoma induction, significantly increased the total number of tumor-infiltrating cytotoxic NK1.1+NK cells (*p* < 0.05; Figure 2(a)). The significantly higher number of IFN-*γ*-producing (*p* < 0.05; Figure 2(b)) and FasL- and granzyme B-expressing (*p* < 0.05; Figures 2(c) and 2(d)) NK cells in the tumors of B16F10+MSC^1d^-treated mice indicated that MSCs, injected 24 h after melanoma induction, enhanced cytotoxic and antitumorigenic potential of NK cells in tumor-bearing animals.

A significantly higher number of CD4+T helper (*p* < 0.05; Figure 2(e)) and CD8+CTLs (*p* < 0.05; Figure 2(i)) were present in the tumors of B16F10+MSC^1d^-treated mice than in melanomas of B16F10+PBS^1d^-treated animals. Phenotype and function of CD4+T helper and CD8+CTLs revealed that MSCs, injected 24 h after melanoma induction, significantly increased the presence of antitumorigenic and IFN-*γ*- and TNF-*α*-producing CD4+Th1 cells (*p* < 0.05 for IFN-*γ*, Figure 2(f); *p* < 0.001 for TNF-*α*, Figure 2(g)), IL-17-producing CD4+Th17 cells (*p* < 0.001, Figure 2(h)), and IFN-*γ*- and TNF-*α*-producing CD8+CTLs (*p* < 0.001, Figures 2(j) and 2(k)) in melanoma-bearing animals.

In line with these findings, significantly higher plasma levels of inflammatory and antitumorigenic cytokines TNF-*α* (*p* < 0.05, Figure 2(l)) and IFN-*γ* (*p* < 0.05, Figure 2(m)) and significantly lower plasma levels of immunosuppressive cytokines TGF-*β* (*p* < 0.05, Figure 2(n)) and IL-10 (*p* < 0.05, Figure 2(o)) were observed in B16F10+MSC^1d^-treated mice, indicating that MSCs, transplanted during the initial phase of melanoma growth, enhanced antitumor immune response in melanoma-bearing animals.

### 3.3. MSCs, Injected 14 Days after Melanoma Induction, Promoted Tumor Growth by Suppressing Antigen-Presenting Properties of Tumor-Infiltrating DCs and Macrophages and by Reducing Tumoricidal Capacity of NK Cells and T Lymphocytes

**Figure 3:**
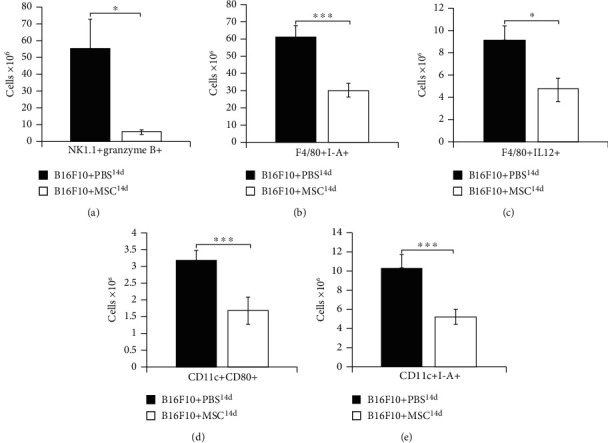
MSCs, injected 14 days after melanoma induction, promoted tumor growth by suppressing cytotoxicity of NK cells and by reducing antigen-presenting properties of tumor-infiltrating macrophages and DCs. Significantly lower number of granzyme B-expressing NK1.1+ cells (a), I-A-expressing and IL-12-producing F4/80+macrophages (b, c), and CD80- and I-A-expressing CD11c+DCs (d, e) were observed in the tumors of B16F10+MSC^14d^-treated mice compared to the B16F10+PBS^14d^-treated animals. Values are presented as the mean ± SEM; *n* = 8 mice/group. ^∗^*p* < 0.05, ^∗∗∗^*p* < 0.001.

Compared to the tumors of B16F10+PBS^14d^-treated mice, the significantly lower number of innate immune cells that play an important role in antitumor immunity (cytotoxic NK cells, inflammatory M1 macrophages and DCs) was observed in melanomas of B16F10+MSC^14d^-treated animals. MSCs, transplanted 14 days after melanoma induction, attenuated tumoricidal capacity of NK cells, as evidenced by the lower number of tumor-infiltrating granzyme B-expressing NK1.1+ cells in B16F10+MSCs^14d^-treated mice (*p* < 0.05, Figure 3(a)). The significantly lower number of tumor-infiltrating, I-A-expressing (*p* < 0.001, Figure 3(b)), and IL-12-producing (*p* < 0.05, Figure 3(c)) F4/80+macrophages and CD80- and I-A-expressing CD11c+DCs (*p* < 0.001, Figures 3(d) and 3(e)) indicated that MSCs alleviated capacity of antigen-presenting cells for optimal activation of T cell-driven antitumor immune response.

**Figure 4:**
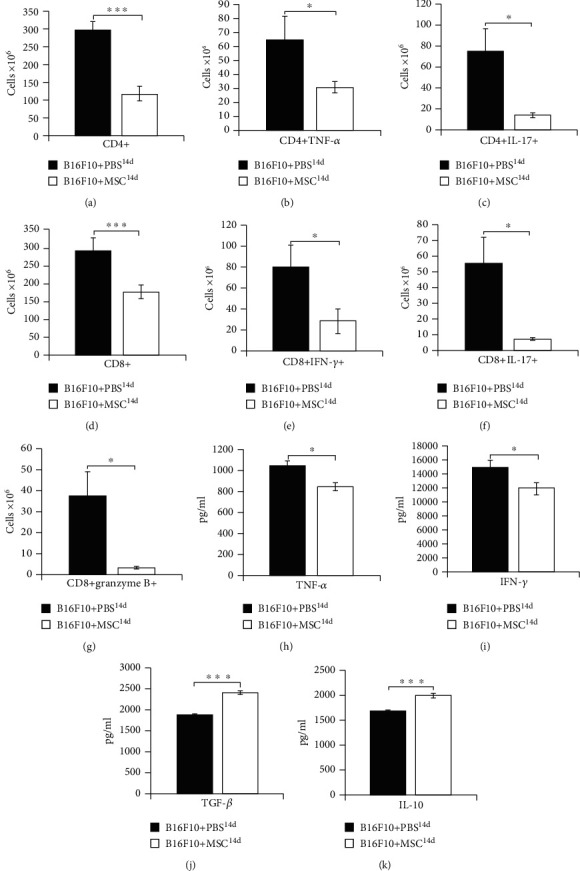
MSCs, injected 14 days after melanoma induction, increased plasma levels of immunosuppressive cytokines and suppressed T cell-driven antitumor immune response in melanoma-bearing animals. Significantly lower number of tumor-infiltrating CD4+T cells (a), TNF-*α* and IL-17-producing CD4+Th1 and Th17 cells (b, c), CD8+CTLs (d), IFN-*γ* and IL-17-producing CD8+CTLs (e, f), granzyme B-expressing CD8+CTLs (g), significantly lower plasma levels of antitumorigenic cytokines TNF-*α* and IFN-*γ* (h, i), and significantly higher plasma levels of immunosuppressive cytokines TGF-*β* and IL-10 (j, k) were noticed in B16F10+MSC^14d^-treated mice compared to the B16F10+PBS^14d^-treated animals. Values are presented as the mean ± SEM; *n* = 8 mice/group. ^∗^*p* < 0.05, ^∗∗∗^*p* < 0.001.

As it is shown in Figure 4, MSCs, injected 14 days after melanoma induction, suppressed tumoricidal capacity of CD8+CTLs, CD4+Th1, and Th17 lymphocytes. Both subpopulations of effector T lymphocytes, CD4+T helper cells (*p* < 0.001, Figure 4(a)) and CD8+CTLs (*p* < 0.001, Figure 4(d)), were significantly reduced in the melanomas of B16F10+MSC^14d^-treated mice compared to B16F10+PBS^14d^-treated animals. Intracellular staining revealed that MSCs suppressed production of tumoricidal cytokines (IFN-*γ* and IL-17) in CD4+Th1 and Th17 cells (*p* < 0.05 for TNF-*α* and IL-17, Figures 4(b) and 4(c)) and in CTLs (*p* < 0.05 for IFN-*γ* and IL-17, Figures 4(e) and 4(f)) of B16F10+MSC^14d^-treated mice, preventing generation of optimal TNF-*α*, IFN-*γ*, and IL-17-driven antitumor immune response. Additionally, a significantly lower number of granzyme B-expressing CD8+CTLs were observed in the tumors of B16F10+MSC^14d^-treated mice (*p* < 0.05, Figure 4(g)), indicating that MSCs injected 14 days after tumor induction significantly reduced the presence of cytotoxic and proapoptotic CD8+CTLs in the tumors of melanoma-bearing animals.

Furthermore, significantly lower levels of antitumorigenic cytokines TNF-*α* and IFN-*γ* (*p* < 0.05, Figures 4(h) and 4(i)) and significantly higher levels of TGF-*β* and IL-10 (*p* < 0.001, Figures 2(j) and 2(k)) were noticed in the plasma samples of B16F10+MSC^14d^-treated mice, indicating that MSCs, injected during the progressive stage of melanoma development, attenuated antitumor immunity by increasing production of immunosuppressive cytokines in tumor-bearing animals.

## 4. Discussion

It is well known that exogenously administered MSCs could migrate to the tumor site where it regulates tumor growth and progression by modulating antitumor immune response [19]. Opposite findings, demonstrating a pro- or anticancer action of transplanted MSCs, were reported in different experimental studies [9–11]. While several research groups revealed that MSCs increased tumor progression [9–11]; results presented in other animal studies showed that transplantation of MSCs led to the alleviation of tumor growth [12–14]. Herewith, we demonstrated that MSCs exert opposite, anti- or protumorigenic action, in the different stages of melanoma progression. MSCs injected in the initial phase of melanoma growth showed a tumor-suppressive effect, while MSCs, administered in the progressive stage of melanoma development, significantly enhanced tumor growth and expansion (Figure 1). In line with our findings are results obtained by Zong and colleagues which are showing that MSC-based effects on progression and metastasis of HCC depend on the stage of cancer development [14]. Although MSCs exhibit tumor-inhibitory effects in the initial phase of HCC development, potent suppression of antitumor immunity accompanied by enhanced HCC progression and metastasis is observed in HCC-bearing rats that received MSCs in the progressive stage of tumor growth [14]. MSCs injected in the initial stage of HCC progression engraft in the microenvironment with the reduced expression of proinflammatory cytokines, while MSCs injected in the progressive phase of HCC growth are exposed to the high levels of inflammatory cytokines [14]. According to the conclusion of Zong and coworkers, the interactions between transplanted MSCs and tumor microenvironment and diverse outcomes of MSC-based therapy depend on the strength of local and systemic inflammatory response during the different phases of hepatocarcinogenesis [14].

Several lines of evidence demonstrated that the ratio between inflammatory and anti-inflammatory cytokines in the microenvironment to which MSCs are exposed directly affects their phenotype and function [18, 20, 21]. When MSCs engraft in the tissue with low levels of inflammatory cytokines and high levels of immunosuppressive cytokines, they adopt proinflammatory (MSC1) phenotype, becoming capable of eliciting potent inflammatory response [20]. On the contrary, MSCs exposed to the high concentration of inflammatory cytokines develop immunosuppressive (MSC2) phenotype, produce a large number of anti-inflammatory factors, and inhibit immune response [18, 20, 21].

Dynamic balance of pro- and anti-inflammatory cytokines within tumor microenvironment regulates melanoma growth and progression [22]. Melanoma cell-derived immunosuppressive cytokines TGF-*β* and IL-10 play a crucially important role in the process of tumor initiation [23–25]. Tumor cell-derived TGF-*β* acts on CTLs to specifically repress the expression of perforin, granzyme B, and FasL and to reduce the production of IFN-*γ*, resulting in a significant attenuation of CTL-mediated tumor cytotoxicity [26]. Through the production of IL-10, melanoma cells prevent maturation of DCs, suppress production of Th1-inducing cytokine IL-12 in DCs, and inhibit their antigen-presenting properties, attenuating generation of effector CD4+Th1 and CD8+CTLs [27]. Furthermore, melanoma cell-primed DCs produce large amounts of immunosuppressive cytokines and significantly contribute to the development of immunosuppressive microenvironment that favors enhanced tumor growth and progression [23–25]. Excessive proliferation of melanoma cells activates stromal and melanoma-residing immune cells (macrophages, DCs, NK, and NKT cells) which produce a large amount of proinflammatory chemokines and cytokines (TNF-*α*, IL-12, IFN-*γ*, and IL-17) that facilitate the massive influx of circulating leucocytes in the tumors and enable generation and expansion of tumorotoxic CD8+CTLs, CD4+Th1, and Th17 cells in the peripheral lymph organs [28]. Generation of potent antitumor immune response during the progressive phase of melanoma growth results in the development of local and systemic inflammation that attenuates melanoma progression [22]. In line with these findings, we assume that changes in the balance between pro- and anti-inflammatory cytokines at the time of MSC administration (initial versus progressive stage of melanoma growth) were crucially responsible for the generation of inflammatory (MSC1) or immunosuppressive (MSC2) phenotype in MSCs after their engraftment in the melanoma-bearing mice (Figure 1).

MSC1, generated in the immunosuppressive microenvironment, produce lymphocyte-attracting chemokines (CCL5, CXCL9, and CXCL10) that recruit CTLs and NK cells to the sites of injury and inflammation [20]. Additionally, MSC1 enhance NK and T cell-dependent antitumor immune response by increasing production of proinflammatory cytokines (TNF-*α*, IFN-*γ*, and IL-17) in these cytotoxic cells [18]. NK cells and CTLs, in a FasL, perforin, and granzyme B-dependent manner, induce apoptosis of melanoma cells [29, 30], while, in an IFN-*γ* and IL-17-dependent manner, enhance antigen-presenting properties of DCs and proinflammatory properties of tumor-infiltrating neutrophils, contributing to the generation of the strong antitumor immune response [31, 32]. In line with these findings, we assume that MSC transplanted in the immunosuppressive microenvironment of B16F10+MSC^1d^-treated animals acquired MSC1 phenotype. MSC1 cells inhibited production of immunosuppressive cytokines (IL-10 and TGF-*β*) in tumor-infiltrating immune cells and promoted generation and influx of cytotoxic, FasL, perforin, and granzyme B-expressing and IFN-*γ*- and IL-17-producing CTLs and NK cells in the tumors of B16F10+MSC^1d^-treated mice which resulted in reduced melanoma growth and progression (Figure 2).

In contrast to the B16F10+MSC^1d^-treated animals, MSCs that were transplanted in B16F10+MSC^14d^-treated mice were exposed to the higher levels of inflammatory cytokines (Figure 1) and generated immunosuppressive, MSC2 phenotype. MSC2, in an IL-10 and TGF-*β*-dependent manner, inhibit maturation of DCs and reduce production of inflammatory cytokines and expression of costimulatory and major histocompatibility class (MHC) II molecules on DCs and macrophages, attenuating their antigen-presenting properties [33]. In line with these findings, we observed a significantly lower number of CD80 and I-A-expressing DCs and reduced presence of IL-12-producing and I-A-expressing macrophages in the tumors of B16F10+MSC^14d^-treated mice compared to B16F10+PBS^14d^-treated animals (Figure 3). MSC2-mediated alleviation of antigen-presenting capacity of tumor-infiltrating DCs resulted in unoptimal activation of naïve CD4+ and CD8+T lymphocytes which led to the reduced presence of effector CD4+Th1 and Th17 cells and CD8+CTLs in the tumors of B16F10+MSC^14d^-treated mice (Figure 4). The reduced number of tumor-infiltrating CTLs, Th1, and Th17 cells corresponded to the increased plasma levels of TGF-*β* and IL-10 in B16F10+MSC^14d^-treated animals, indicating the important role of TGF-*β* and IL-10 in MSC-mediated suppression of T cell-driven antitumor immune response. It is well known that MSC2, through the production of TGF-*β* and IL-10, directly suppress activation of the Jak-Stat signaling pathway in proliferating T lymphocytes, causing the G1 cell cycle arrest [34]. Additionally, MSC2-sourced TGF-*β* and IL-10 downregulate production of inflammatory cytokines and reduce cytotoxicity of Th1 and Th17 cells and CTLs, contributing to the enhanced tumor growth and progression [35, 36]. Therefore, we believe that MSCs that were injected in B16F10-treated mice during the progressive stage of melanoma growth adopted immunosuppressive MSC2 phenotype and in a TGF-*β* and IL-10-dependent manner attenuated antitumor immune response, resulting in increased melanoma growth.

**Figure 5:**
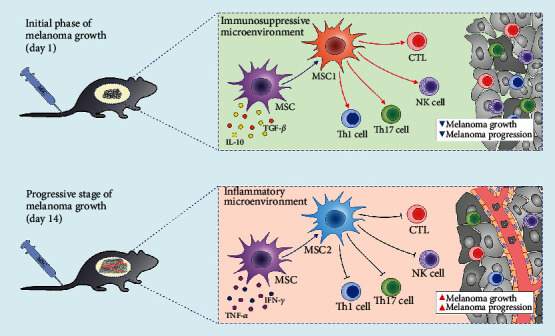
The effects of MSCs on antimelanoma immunity depend on the timing of their administration. MSCs transplanted during the initial phase of melanoma growth exerted tumor-suppressive effects. Since these MSCs were exposed to the immunosuppressive microenvironment (established by IL-10 and TGF-*β*-producing tumor cells), they acquired inflammatory, MSC1 phenotype and induced expansion of cytotoxic NK cells and antitumorigenic CD8+CTLs, CD4+Th1, and Th17 lymphocytes, resulting in attenuated melanoma growth and progression. On the contrary, MSCs transplanted during the progressive stage of melanoma development were exposed to the high concentration of inflammatory cytokines (TNF-*α* and IFN-*γ*) and generated immunosuppressive, MSC2 phenotype. Accordingly, enhanced melanoma growth and reduced number of tumor-infiltrating antigen-presenting cells (macrophages and DCs), cytotoxic CTLs and NK cells, and antitumorigenic CD4+Th1 and Th17 lymphocytes were observed in melanoma-bearing mice which received MSCs during the progressive stage of tumor development.

In summing up, MSCs have an opposite role in the different stages of melanoma progression. MSCs transplanted during the initial phase of melanoma growth exert tumor-suppressive effect, while MSCs injected in the progressive stage of melanoma development suppressed antitumor immunity and enhanced tumor expansion (Figure 5). Therefore, the timing of MSC administration into the tumor microenvironment is crucially important for MSC-dependent modulation of melanoma progression.

## Data Availability

The data used to support the findings of this study are included within the article.

## References

[1] Liu Y., Sheikh M. S. (2014). Melanoma: molecular pathogenesis and therapeutic management. *Molecular and Cellular Pharmacology*.

[2] Jenkins R. W., Fisher D. E. (2020). Treatment of advanced melanoma in 2020 and beyond. *Journal of Investigative Dermatology*.

[3] Volarevic V., Ljujic B., Stojkovic P., Lukic A., Arsenijevic N., Stojkovic M. (2011). Human stem cell research and regenerative medicine--present and future. *British Medical Bulletin*.

[4] Castro-Manrreza M. E. (2016). Participacion de las celulas troncales mesenquimales en la regulacion de la respuesta inmune y el desarrollo de cancer. *Boletín Médico del Hospital Infantil de México*.

[5] Kidd S., Spaeth E., Dembinski J. (2009). Direct evidence of mesenchymal stem cell tropism for tumor and wounding microenvironments using in vivo bioluminescent imaging. *Stem Cells*.

[6] Poggi A., Varesano S., Zocchi M. R. (2018). How to hit mesenchymal stromal cells and make the tumor microenvironment immunostimulant rather than immunosuppressive. *Frontiers in Immunology*.

[7] Dwyer R. M., Potter-Beirne S. M., Harrington K. A. (2007). Monocyte chemotactic protein-1 secreted by primary breast tumors stimulates migration of mesenchymal stem cells. *Clinical Cancer Research*.

[8] Galland S., Stamenkovic I. (2020). Mesenchymal stromal cells in cancer: a review of their immunomodulatory functions and dual effects on tumor progression. *The Journal Of Pathology*.

[9] De Boeck A., Pauwels P., Hensen K. (2013). Bone marrow-derived mesenchymal stem cells promote colorectal cancer progression through paracrine neuregulin 1/HER3 signalling. *Gut*.

[10] Karnoub A. E., Dash A. B., Vo A. P. (2007). Mesenchymal stem cells within tumour stroma promote breast cancer metastasis. *Nature*.

[11] Hernanda P. Y., Pedroza-Gonzalez A., van der Laan L. J. (2013). Tumor promotion through the mesenchymal stem cell compartment in human hepatocellular carcinoma. *Carcinogenesis*.

[12] Yulyana Y., Ho I. A., Sia K. C. (2015). Paracrine factors of human fetal MSCs inhibit liver cancer growth through reduced activation of IGF-1R/PI3K/Akt signaling. *Molecular Therapy*.

[13] Lee R. H., Yoon N., Reneau J. C., Prockop D. J. (2012). Preactivation of human MSCs with TNF-*α* enhances tumor-suppressive activity. *Cell Stem Cell*.

[14] Zong C., Zhang H., Yang X. (2018). The distinct roles of mesenchymal stem cells in the initial and progressive stage of hepatocarcinoma. *Cell Death & Disease*.

[15] Potez M., Fernandez-Palomo C., Bouchet A. (2019). Synchrotron microbeam radiation therapy as a new approach for the treatment of radioresistant melanoma: potential underlying mechanisms. *International Journal of Radiation Oncology • Biology • Physics*.

[16] Volarevic V., Markovic B. S., Jankovic M. G. (2019). Galectin 3 protects from cisplatin-induced acute kidney injury by promoting TLR-2-dependent activation of IDO1/Kynurenine pathway in renal DCs. *Theranostics*.

[17] Ngiow S. F., von Scheidt B., Möller A., Smyth M. J., Teng M. W. L. (2013). The interaction between murine melanoma and the immune system reveals that prolonged responses predispose for autoimmunity. *Oncoimmunology*.

[18] Gazdic M., Volarevic V., Arsenijevic N., Stojkovic M. (2015). Mesenchymal stem cells: a friend or foe in immune-mediated diseases. *Stem Cell Reviews and Reports*.

[19] Lin W., Huang L., Li Y. (2019). Mesenchymal stem cells and cancer: clinical challenges and opportunities. *BioMed Research International*.

[20] Li W., Ren G., Huang Y. (2012). Mesenchymal stem cells: a double-edged sword in regulating immune responses. *Cell Death & Differentiation*.

[21] Waterman R. S., Tomchuck S. L., Henkle S. L., Betancourt A. M. (2010). A new mesenchymal stem cell (MSC) paradigm: polarization into a pro-inflammatory MSC1 or an immunosuppressive MSC2 phenotype. *PLoS One*.

[22] Neagu M., Constantin C., Caruntu C., Dumitru C., Surcel M., Zurac S. (2018). Inflammation: a key process in skin tumorigenesis. *Oncology Letters*.

[23] Busse A., Keilholz U. (2011). Role of TGF*β* in melanoma. *Current Pharmaceutical Biotechnology*.

[24] Wiguna A. P., Walden P. (2015). Role of IL-10 and TGF-*β* in melanoma. *Experimental Dermatology*.

[25] Rabinovich G. A., Gabrilovich D., Sotomayor E. M. (2007). Immunosuppressive strategies that are mediated by tumor cells. *Annual Review of Immunology*.

[26] Thomas D. A., Massagué J. (2005). TGF-*β* directly targets cytotoxic T cell functions during tumor evasion of immune surveillance. *Cancer Cell*.

[27] Gerlini G., Tun-Kyi A., Dudli C., Burg G., Pimpinelli N., Nestle F. O. (2004). Metastatic melanoma secreted IL-10 down-regulates CD1 molecules on dendritic cells in metastatic tumor lesions. *The American Journal of Pathology*.

[28] Passarelli A., Mannavola F., Stucci L. S., Tucci M., Silvestris F. (2017). Immune system and melanoma biology: a balance between immunosurveillance and immune escape. *Oncotarget*.

[29] Leignadier J., Favre S., Luther S. A., Luescher I. F. (2015). CD8 engineered cytotoxic T cells reprogram melanoma tumor environment. *OncoImmunology*.

[30] Zhu L., Kalimuthu S., Gangadaran P. (2017). Exosomes derived from natural killer cells exert therapeutic effect in melanoma. *Theranostics*.

[31] Ni L., Lu J. (2018). Interferon gamma in cancer immunotherapy. *Cancer Medicine*.

[32] Chen Y. S., Huang T. H., Liu C. L. (2019). Locally targeting the IL-17/IL-17RA axis reduced tumor growth in a murine B16F10 melanoma model. *Human Gene Therapy*.

[33] Harrell C. R., Jankovic M. G., Fellabaum C. (2019). Molecular mechanisms responsible for anti-inflammatory and immunosuppressive effects of mesenchymal stem cell-derived factors. *Advances in Experimental Medicine and Biology*.

[34] Bright J. J., Kerr L. D., Sriram S. (1997). TGF-beta inhibits IL-2-induced tyrosine phosphorylation and activation of Jak-1 and stat 5 in T lymphocytes. *The Journal of immunology*.

[35] Volarevic V., Gazdic M., Simovic Markovic B., Jovicic N., Djonov V., Arsenijevic N. (2017). Mesenchymal stem cell-derived factors: immuno-modulatory effects and therapeutic potential. *BioFactors*.

[36] García-Rocha R., Moreno-Lafont M., Mora-García M. L. (2015). Mesenchymal stromal cells derived from cervical cancer tumors induce TGF-*β*1 expression and IL-10 expression and secretion in the cervical cancer cells, resulting in protection from cytotoxic T cell activity. *Journal of Translational Medicine*.

